# Assembly and use of a low-cost paracentesis simulator for the teaching of puncture and drainage of ascites

**DOI:** 10.1590/0100-6991e-20223099

**Published:** 2022-02-18

**Authors:** BRENO WELLINGTON MESQUITA SILVEIRA, LUANA AKEMI ALVES ARAÚJO, LUCAS DE SOUZA ALBUQUERQUE, FELIPE DE OLIVEIRA VASCONCELOS, ERICK BATISTA DE MEDEIROS LIMA, ANNYA COSTA ARAÚJO DE MACEDO GÓES, LARA BURLAMAQUI VERAS

**Affiliations:** 1 - Universidade Federal do Ceará, Departamento de Cirurgia - Fortaleza - CE - Brasil; 2 - Hospital Universitário Walter Cantídio, Departamento de Cirurgia - Fortaleza - CE - Brasil

**Keywords:** Simulation Training, Low-cost Technology, Paracentesis, Ascites, Education, Medical, Treinamento por Simulação, Tecnologia de Baixo Custo, Paracentese, Ascite, Educação Médica

## Abstract

**Objectives:**

to describe the assembly of a low-cost paracentesis simulator and evaluate its effectiveness, acceptance and impact on the learning of medical students.

**Methodology:**

a paracentesis simulator was built using a mannequin and materials such as plastic bottles, Velcro, polyvinyl chloride sheets and silicone were used. A cross-sectional and experimental study was carried out with undergraduate medical students without previous practical experience with paracentesis, which sought to validate the model, evaluating its benefits in learning and obtaining technical skills.

**Results:**

after using the simulator there was an increase of 82.4% in the level of confidence in performing paracentesis in a patient, with 98% of respondents considering that the model fulfilled the simulator function with satisfaction, and 100% considering it useful as a teaching tool.

**Conclusion:**

the built simulator was effective as an educational resource, serving as an alternative to high-cost commercial models, allowing for greater accessibility in the use of this tool in medical education.

## RESUMO

**Objetivos:**

descrever a montagem de simulador de paracentese de baixo custo e avaliar eficácia, aceitação e impacto no aprendizado de acadêmicos do curso de medicina.

**Método:**

um simulador de paracentese foi construído a partir de manequim e de materiais como garrafas plásticas, velcro, folhas de policloreto de vinila e silicone. Foi feito estudo transversal com estudantes da graduação do curso de medicina sem experiência real prévia com paracentese, que buscou validar o modelo, por meio da análise de benefícios no aprendizado e na obtenção de habilidades técnicas.

**Resultados:**

após o uso do simulador, observou-se aumento de 82,4% no nível de segurança na realização da paracentese em paciente, 98% dos pesquisados consideraram que o uso do modelo cumpriu com satisfação a função de simulador e 100% definiram-no útil como ferramenta de ensino.

**Conclusão:**

a montagem do simulador mostrou-se factível e eficaz como recurso educacional. Serviu como alternativa aos modelos comerciais de alto custo e permitiu maior acessibilidade do uso dessa ferramenta na educação médica.

## INTRODUCTION

Paracentesis is an intervention that consists of drainage of ascitic fluid by puncture with a catheter, under local anesthesia. The motivation for this procedure can be diagnostic or therapeutic. In the first case, it is useful to define behavior and, in the second, essential to alleviate symptoms^1^ ^-^ ^3^.

Due to the varied etiology and high incidence of patients with ascites in medical services, the technique of paracentesis must be well-founded by medical professionals to avoid complications arising from execution errors^2^ ^-^ ^4^.

A simulation consists of practical experience, reproduced in a controlled environment, which allows training and prepares the student for a given real situation. Currently, simulation is increasingly recognized as a tool for learning and improving practices that encompass medical education. Training and repeating a certain task enables and provides the development of executing skills, in addition to working on other competencies that are important for the trainee physician, such as communication, leadership and teamwork. Furthermore, this path allows active learning in an environment free of biological risks and minimizes the use of animal guinea pigs^5^ ^-^ ^10^.

As a tool already consolidated in the teaching of the health area, especially in medicine, the use of simulators led to the term Simulation-Based Teaching (EBS). The EBS is a very widespread resource in courses and routine assessments, with positive effects on the learning curve and on the acquisition of skills, as it directs the student’s attention to the step-by-step procedure^3^ ^,^ ^9^ ^,^ ^10^.

Obtaining industrialized simulators is costly. This makes the acquisition often unfeasible for several educational institutions, especially those with low economic investment. Therefore, the creation of affordable cost models became an advantageous alternative by having simulation models as an instrument of greater accessibility and, thus, enabling and expanding the use of this resource by the academic public^3^ ^-^ ^7^ ^,^ ^9^ ^-^ ^11^.

The aim of this article is to describe the assembly of a low-cost paracentesis simulator and evaluate its effectiveness, acceptance and impact on the learning of medical students.

## METHODS

### Simulator construction

**Figure 1:**
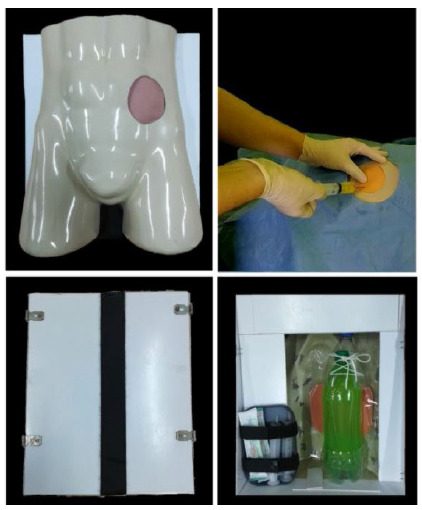
Pre-simulation front view (top left figure), vision during aspiration (top right figure), dorsal external view (bottom left figure), internal dorsal view (bottom right figure).

The simulator was made from the following materials: male hip dummy, polyethylene terephthalate (PET) bottles, 3mm thick polyvinyl chloride (PVC) sheets, 15x11cm silicone, Velcro, syringes and 14G intravenous catheter (Figure 1). Different materials were tested during the construction of the simulator, these being chosen for their characteristics of low cost, easy availability and aesthetic and tactile equivalence.

It started with an orifice of approximately 11cm in diameter in the topography of the left iliac fossa of the manikin. In the dorsal region of the mannequin, a box made of cutouts and glued PVC sheets was built. In order to create the lid, two pieces of PVC were screwed onto the sides of the mannequin, on each piece there were two metal hinges. On the medial edges of each side of the two pieces, 2cm thick Velcro tapes were placed to allow opening and closing the access to the internal cavity of the mannequin.

**Figure 2:**
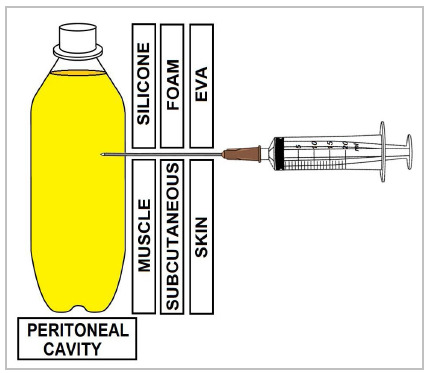
Simulator layer scheme and comparison between simulator and abdominal wall layers.

Then, a 1L bottle filled with water colored with yellow gouache paint was used in order to simulate the peritoneal cavity and ascitic fluid, respectively. This was mounted on an internal bracket made of cutouts from the ends of a 2L plastic bottle. The face of the bottle facing the orifice was sanded in order to reduce the thickness of the plastic and improve the reliability of the punch. Between the bottle and the orifice, a piece of silicone was placed, first, in order to simulate muscle tissue, and, second, a 1cm thick D28 foam coated with ethylene vinyl acetate (EVA) sheet. to simulate the skin and absorb the anesthetic. The total cost of the simulator was approximately R$100,00 (one hundred reais) (Figure 2).

### Scientific method

A cross-sectional study was carried out to evaluate the development and application of a low-cost simulator, developed by medical students, for training in paracentesis.

The inclusion criteria were: being a medical graduate student, regardless of the educational institution, semester or previous experience, real or simulated, in paracentesis. Exclusion criteria were: students who did not attend the study site on the scheduled day.

The study was carried out with a non-probabilistic sample. Participants were invited, voluntarily, through an online form, which included identification questions (name, age, gender and semester), previous experience of theoretical classes, use of simulators and real experience with the paracentesis procedure. The research had the initial enrollment of 69 participants, however, 18 of them were excluded from the study due to non-attendance on the informed day and time, resulting in a final number of 51 individuals.

The research was divided into 4 stages: (1) application of a pre-test questionnaire, (2) theoretical class, (3) simulation with application of a checklist and (4) application of a post-test questionnaire.

The pre-test questionnaire was composed of a single question related to the level of safety in performing the paracentesis procedure in a patient, which was later repeated in the post-test questionnaire. Participants should answer with “yes” or “no” and inform the degree of security or insecurity (little, medium or high).

After the pre-test, there was a brief theoretical class on paracentesis, which included indications, technique and explanatory video, in addition to practical concepts of antisepsis and anesthesia.

At the end of the class, the students went to a room to carry out the simulation. On site, there was a bench, where the simulator and the materials needed for the procedure were found: procedure gloves, antiseptic, tub, forceps with gauze, 5mL syringe filled with anesthetic attached to a 22G venous catheter, 20mL empty syringe attached to 14G venous catheter and field cloth. The anesthetic was simulated by water, the antiseptic by water dyed with orange food dye, sterilized gloves by procedure gloves and the procedure gloves by degloved hands (the candidate was previously informed about these substitutions).

There was also a fictitious clinical case that contextualized the indication of diagnostic paracentesis and an evaluator who instructed the participant on the use of the simulator and the time of five minutes available to complete the activity.

During the execution of the simulation, a checklist, developed by the researchers, was applied, which evaluated nine steps, each classified as correctly performed (RC), incorrectly performed (RI) and not performed (NR). The steps and criteria for RC and RI are described in table I. The start of the simulation occurred after the evaluator’s verbal authorization and the end was considered in two situations: (1) the end of the time period and (2) when the candidate, after aspiration of ascitic fluid, removed and capped the catheter within the time period. After completing the task, the evaluator clarified the candidate’s mistakes and successes. For cases where the participant exceeded the time period, the evaluator informed the end of the simulation and, in a non-evaluative nature, assisted and allowed the conclusion. It is noteworthy that the participant could only interact with the evaluator when the questions were related to stages I and II.

**Table 1:** Criteria for evaluating the checklist.

Stage		RC	RI
I	Presentation and explanation of the procedure for the patient	The participant must say the name and title, ask the patient's name and explain the procedure to be performed in intelligible language	Absence of any of these steps, but not all
II	Questioning about full bladder and request for voiding	The participant must ask if the patient is in the mood to urinate and, if so, must ask him to urinate before the procedure	Absence of any of these steps, but not all
III	Identification of the puncture site	The participant must locate the puncture point, at the intersection between the middle and distal third of the imaginary line between the umbilical scar and the left anterior superior iliac spine	Identification of a site more proximal or distal to the desired point or any other non-matching anatomical site
IV	Wearing gloves	The participant must wear sterilized gloves immediately before antisepsis	He wore gloves only after antisepsis, before locating the puncture point, or he wore them and kept them on inappropriately
V	Antisepsis	With gloves on, dip gauze into the tub with antiseptic and apply antiseptic in circular and centrifugal movements in relation to the puncture point	Apply antiseptic degloved, in non-circular or centripetal movements, breaking the antiseptic technique due to contamination of the gloves
VI	field cloth	With gloves on, the participant must open the field cloth and position the fenestra to isolate the puncture point. This step must be performed between antisepsis and anesthesia	Placement of the field cloth before antisepsis or after anesthesia or degloved
VII	Anesthesia	Apply infiltrative local anesthetic at the puncture point, penetrate at an acute angle with a 5mL syringe coupled to a 22G intravenous catheter with a bevel oriented upwards, then penetrate into the deep planes at a more perpendicular angulation and anesthetize the path, always aspirating before the applications	No aspiration before applications, penetration with downward bevel orientation, no anesthesia of the dermis or puncture path, penetration with incorrect angulation and no puncture pressure control
VIII	Puncture technique	Perform Z-traction technique or skin traction with catheter, puncture at the appropriate point with a 20mL syringe coupled to a 14G intravenous catheter, with bevel oriented upwards, always aspirating and performing penetration force control	Absence of skin traction, excessive penetration or incorrect angulation, multiple superficial penetrations
IX	Ascitic fluid aspiration	Aspirate 20mL of ascitic fluid after reaching the peritoneal cavity	Insufficient aspiration, return the aspirated liquid back to the peritoneal cavity

RC: Performed correctly. RI: Performed incorrectly.

Finally, students were submitted to a post-test questionnaire consisting of seven questions, five evaluating the simulator: if it was functional, if it consolidated theoretical learning, if it should be used before actual practice, if it fulfilled the role of an educational tool and whether it should be increased in medical education during graduation. The available options were: Strongly Agree (CT), Partially Agree (CP), Indifferent (I), Partially Disagree (DP) and Strongly Disagree (DT). The sixth question asked about the stage of the simulation in which the participant faced greater difficulty. The seventh returned to the question about the safety of performing the procedure on a patient.

This study was submitted and approved by the Research Ethics Committee of the Federal University of Ceará (CEP/UFC/PROPESQ) under protocol number 4.143.346.

### Statistical analysis

The data obtained were computed and analyzed using programs such as Microsoft Excel^®^ and GraphPad Prism9^®^, using the t test with paired samples. The rejection of the null hypothesis was established for values of p<5%.

 To calculate the sample, the G-Power 3.1.9.2 program was used, in which the following was found: sample power of 0.8, significance level 0.05 and effect size 0.5. The minimum sample found was 35 people, but it was decided to recruit 51 students in order to contemplate losses. The difference between the students’ responses was assessed using the Wilcoxon W test and the normality of statistical data was estimated using the Shapiro-Wilk test (0.8 p<0.001).

## RESULTS

The research was attended by 51 medical students, of which 25 (49.0%) were female and 26 (51.0%) were male. The age of respondents ranged between 18 and 41 years for females with an average of 23.6 years and between 18 and 38 years for males with an average of 23.7 years. The research was carried out with students from the 1^st^ to the 10^th^ period, with 56.9% of them belonging to the 1^st^ to the 4^th^ period and 41.2% belonging to the 5^th^ to the 8^th^ period. In addition, 15.7% had previous experience with some type of simulator and 45.1% had had some theoretical class on the topic of paracentesis.

In the question “Do you feel safe to perform a paracentesis in a patient?”, 46 out of 51 respondents (90.2%) stated that they were initially unsafe. After the simulation, 42 out of 46 unsafe individuals stated that they had acquired confidence in performing the procedure (which represents an increase of 82.34% in the safety index, p<0.0001). At the end of the simulation, 92.2% achieved some degree of security, with 68.6% of those considered medium to very safe (Table 2).

**Table 2:** Safety for performing paracentesis in a real situation.

	Not			Yes		
	MINS	MEINS	PINS	PSEG	MESEG	MUSEG
Before the Simulation	54,9%	31,3%	3,9%	3,9%	5,9%	0%
After Simulation	3,9%	0%	3,9%	23,5%	62,7%	5,9%

MINS: Muita insegurança (a lot of insecurity). MEINS: Média insegurança (medium insecurity). PINS: Pouca insegurança (little insecurity). PSEG: Pouca segurança (little security). MESEG: Média segurança (medium security). MUSEG: Muita segurança (a lot of security)

The assessment of the difference in the level of security before and after using the simulator was estimated using the Wilcoxon W test, after stipulation of a numerical value from 1 to 6 for the security categories, 1 MINS and 6 MUSEG. Thus, a significant difference was found (p<0.001), in which the post-simulation was greater than the pre-simulation.

Regarding the post-test, 50 individuals (98.0%) agreed that the model fulfilled the role of simulator with satisfaction and 51 (100%) agreed that the simulator would be useful as an educational tool, which consolidated the theoretical learning, which should be used as training before the real situation and that should be increased as a practical activity during medical graduation (Table 4).

**Table 3:** Test of Wilcoxon W (p<0,001).

	N	Mean	Median	SD	SE
pre-simulation	51	1,75	1	1,11	0,16
post-simulation	51	4,59	5	0,96	0,13

**Table 4:** Test of Wilcoxon W (p<0,001).

	DT	DP	I	CP	CT
Did the created model fulfill the role of simulator with satisfaction?	0%	0%	1 (1,9%)	13 (25,5%)	37 (72,6%)
Is the simulator useful as an educational tool?	0%	0%	0%	3 (5,9%)	48 (94,1%)
Does the simulation consolidate theoretical learning?	0%	0%	0%	4 (7,8%)	47 (92,2%)
Should the simulator be used as a training method before the real situation?	0%	0%	0%	1 (1,9%)	50 (98,1%)
Should the simulator be enhanced during medical graduation?	0%	0%	0%	1 (1,9%)	50 (98,1%)

DT: Discordo totalmente (I totally disagree). DP: Discordo parcialmente (I partially disagree). I: Indiferente (indifferent). CP: Concordo parcialmente (partially agree). CT: Concordo totalmente (I totally agree).

Regarding the simulation stage in which the participants thought they had greater difficulty, the citation of more than one stage per participant was allowed, so there were a total of 79 responses. In this sense, 43.1% of the participants highlighted the puncture technique as the greatest difficulty, 29.4% claimed to be the aspiration of the ascitic content, 31.4% the performance of anesthesia, 19.6% the performance of antisepsis, 13.7% the placement of the field cloth, 9.8% the presentation and explanation of the procedure to the patient and 9.8% the identification of the puncture site.

About 78.4% managed to complete the practice on time. The average time to complete the simulation was 4 minutes and 3 seconds.

Regarding the results of the checklist, there was a higher RC index in stages I, III, V, VI, VIII and IX, while RI prevailed in stages IV and VII and NR in stage II (Table 5).

**Table 5:** Checklist results.

STAGE	NR	RI	RC
I	17,6%	3,9%	78,4%
II	58,8%	0%	41,2%
III	31,4%	0%	68,6%
IV	1,9%	70,6%	27,5%
V	15,7%	4,0%	80,3%
VI	17,6%	11,8%	70,6%
VII	3,9%	51,0%	45,1%
VIII	3,9%	47,0%	49,0%
IX	21,6%	7,8%	70,6%

## DISCUSSION

The use of simulators has been increasingly important as a tool for medical education as they bring students closer to professional reality. In addition, there is a growing demand from society for greater safety in surgical procedures and better training for physicians^3^ ^,^ ^12^. However, the training of undergraduates, through simulators, is made difficult due to the high market value of these products, which makes it impossible to purchase by institutions with smaller funds and, even when present in reference education centers, there may be certain restrictions use by academics, such as exclusive access to inmates and residents^3^ ^,^ ^11^ ^,^ ^17^. Furthermore, due to the current coronavirus pandemic scenario, interactions between students and patients have become restricted. Thus, practical classes, in skill laboratories, with the use of simulators in a controlled environment, is an option that can, at least partially, meet the educational needs.

This situation supports the need to develop accessible and effective simulators for learning, which can be manufactured without major difficulties, whether by students, academic leagues, faculty or technical assistants. This allows any institution to take advantage of this complementary method.

The effectiveness of the simulator in question had an excellent evaluation, since all respondents agreed that the model fulfilled the simulator function and 100% stated that it was useful as an educational tool.

The fact that everyone agrees that simulation consolidates theoretical learning, added to the gain in carrying out the procedure, strongly indicates the benefit of this tool. The literature points to the need to carry out practical activities to acquire skills, especially in surgical and interventional medicine areas, something that purely observational or theoretical learning does not offer^14^ ^,^ ^15^.

It is worth noting that the training carried out contemplates the complete technique of performing the paracentesis, so that it adds skills and knowledge of antisepsis, anesthesiology and interaction with the patient illustrated by the clinical case. Thus, the built model has the potential to prepare the student for the real experience, which constitutes the objective of active methods of the EBS^5^ ^,^ ^16^.

Another notable aspect of the simulator is precisely the possibility of the undergraduate training repeatedly to perform the procedure before performing it on a real patient, if necessary. Thus, the student uses a teaching method similar to strategies based on the spaced repetition system (SRE), which is a method based on the forgetting curve and on the fact that there is an ideal time to review what is learned^8^ ^,^ ^11^ ^,^ ^17^. Thus, with each new attempt, the student acquires more experience and improves their skills, which increases, with each repetition, the learning curve. These aspects were reflected in the survey results, in which all participants highlighted the importance of using simulators before a real situation and the need for this equipment during medical training.

As limitations of the research carried out, the low percentage of students who had previous experience with simulators and theory on the subject is highlighted. Furthermore, the fact that most participants were at the beginning of their medical training (more than half between the 1^st^ and 4^th^ periods), when the subjects of surgery, anesthesiology and gastroenterology were not yet covered in the classroom. Consequently, this public was presented with completely new knowledge. The diversity of the participants’ academic periods is also included, which can lead to variations in knowledge, an aspect capable of directly influencing the research results. Thus, in order to obtain more reliable results, in future research, participants with greater experience in performing the paracentesis procedure should be selected, either through practice in real situations or in a training scenario with other simulators. In this way, a more truthful comparison can be made with the researched simulator, allowing to verify with greater precision the effectiveness and advantages of the proposed model.

## CONCLUSION

Low-cost simulators are educational tools with the potential to provide professionals with skill, technical knowledge and confidence in a given procedure. When used along the lines of the EBS, they become even more effective as they bring the practice closer to reality, and their use should be encouraged and implemented by medical education institutions.
